# Disparities in all-cause mortality among people experiencing homelessness in Toronto, Canada during the COVID-19 pandemic: a cohort study

**DOI:** 10.3389/fpubh.2024.1401662

**Published:** 2024-08-09

**Authors:** Lucie Richard, Brooke Carter, Linda Wu, Stephen W. Hwang

**Affiliations:** ^1^MAP Centre for Urban Health Solutions, Unity Health Toronto, Toronto, ON, Canada; ^2^ICES Western, London Health Sciences Research Institute, London, ON, Canada; ^3^Faculty of Health Sciences, McMaster University, Hamilton, ON, Canada; ^4^ICES, Toronto, ON, Canada; ^5^Dalla Lana School of Public Health, University of Toronto, Toronto, ON, Canada; ^6^Department of General Internal Medicine, University of Toronto, Toronto, ON, Canada

**Keywords:** homelessness, mortality, COVID-19 pandemic, health disparities, health services research

## Abstract

People experiencing homelessness have historically had high mortality rates compared to housed individuals in Canada, a trend believed to have become exacerbated during the COVID-19 pandemic. In this matched cohort study conducted in Toronto, Canada, we investigated all-cause mortality over a one-year period by following a random sample of people experiencing homelessness (*n* = 640) alongside matched housed (*n* = 6,400) and low-income housed (*n* = 6,400) individuals. Matching criteria included age, sex-assigned-at-birth, and Charlson comorbidity index. Data were sourced from the *Ku-gaa-gii pimitizi-win* cohort study and administrative databases from ICES. People experiencing homelessness had 2.7 deaths/100 person-years, compared to 0.7/100 person-years in both matched unexposed groups, representing an all-cause mortality unadjusted hazard ratio (uHR) of 3.7 (95% CI, 2.1–6.5). Younger homeless individuals had much higher uHRs than older groups (ages 25–44 years uHR 16.8 [95% CI 4.0–70.2]; ages 45–64 uHR 6.8 [95% CI 3.0–15.1]; ages 65+ uHR 0.35 [95% CI 0.1–2.6]). Homeless participants who died were, on average, 17 years younger than unexposed individuals. After adjusting for number of comorbidities and presence of mental health or substance use disorder, people experiencing homelessness still had more than twice the hazard of death (aHR 2.2 [95% CI 1.2–4.0]). Homelessness is an important risk factor for mortality; interventions to address this health disparity, such as increased focus on homelessness prevention, are urgently needed.

## 1 Introduction

People experiencing homelessness (PEH) have been found to have consistent patterns of poorer health and increased mortality in comparison to housed counterparts over the past several decades (1–8). This pattern persists even after accounting for potential confounding by low socioeconomic status and comorbidities (2, 3, 8). The COVID-19 pandemic has further exacerbated challenges faced by PEH, with direct impacts through elevated infection rates and subsequent adverse outcomes (9–13), as well as indirect impacts through disruptions to essential services (13–17). Furthermore, the pandemic amplified existing mental health and substance use struggles, contributing to a surge in acute mental health events, substance use, and instances of self-harm (13, 18–23). However, in response to the pandemic many cities including Toronto, Canada introduced emergency measures such as temporary shelter hotels to mitigate against high infection risk in congregate settings (13, 24–29), and these may have had the effect of improving housing conditions, at least for those accessing these services. Consequently, it is unclear whether the pandemic has impacted mortality and excess mortality for PEH overall.

Initial reports from the United States suggest the pandemic may have worsened PEH mortality (23) and disparity in mortality between PEH and housed counterparts (24, 30). However, PEH mortality and excess mortality in Canada during the pandemic remains unexplored at this time. This study assesses all-cause mortality over a one-year period during the pandemic among a prospectively followed, representative cohort of PEH in Toronto, Canada. Furthermore, we evaluate excess mortality in this group as compared to matched housed and low-income housed individuals.

## 2 Methods

### 2.1 Study design and setting

We conducted a matched cohort study in Toronto, Canada’s most populous city, using a combination of prospectively collected research study data from the *Ku-gaa-gii pimitizi-win* study and retrospective administrative data held at ICES (31). ICES is an independent, non-profit research institute whose legal status under Ontario’s health information privacy law allows it to collect and analyze health care and demographic data for health system evaluation and improvement. The *Ku-gaa-gii pimitizi-win* study is the largest representative cohort of people experiencing homelessness in Canada during the COVID-19 pandemic, and consists of a random sample of people experiencing homelessness during 2021 and 2022 in Toronto, a city on Treaty 13 territory in Canada. The group characteristics of this cohort closely resembles that of Toronto’s 2021 Point in Time count (32). The *Ku-gaa-gii pimitizi-win* study protocol is available elsewhere (33).

During the observation period, the City of Toronto leased a series of hotels to move thousands of individuals experiencing homelessness out of crowded emergency shelters, deemed too risky for COVID-19 transmission and infection (34). These had the unintentional benefit of improving emergency housing conditions greatly, at least for clients in these hotels. On the other hand, public health measures applied in Ontario restricting availability and access to various services resulted in greatly exacerbated challenges faced by PEH in securing basic needs (13–17). This, in turn amplified existing mental health and substance use struggles in this population, contributing to increases in acute mental health events, substance use, and instances of self-harm (13, 18–23).

Study data, mortality data and other information from health administrative databases were linked using unique encoded identifiers and analyzed at ICES. This study follows the Reporting of Studies Conducted Using Observational Routinely Collected Data (RECORD) reporting guidelines (Supplementary material 1) (35).

### 2.2 Data sources

We leveraged data from the *Ku-gaa-gii pimitizi-win* study to identify the sample of people experiencing homelessness in 2021, which we defined as the exposed group. We also used a number of data sources at ICES to define participants in the unexposed groups as well as the outcomes and covariates for all three groups. These data sources include: the ICES Registered Persons Database (RPDB); the Discharge Abstract Database; the National Ambulatory Care Reporting System database; the Ontario Mental Health Reporting System database; the Ontario Health Insurance Plan (OHIP) claims database; the Community Health Centre database; the Ontario Cancer Registry; and several ICES-derived population-surveillance databases, including the Chronic Obstructive Pulmonary Disease Database, the Ontario Asthma Database, the Ontario Diabetes Database, the Congestive Heart Failure Database, the Ontario Hypertension Database, and the Ontario HIV database. These data sources are further detailed in Supplementary material 2.

### 2.3 Population

We recruited *Ku-gaa-gii pimitizi-win* participants by approaching individuals in randomly selected beds or rooms at 61 participating shelters and physical distancing hotels in Toronto between June 16 and September 9, 2021; participants were additionally recruited from one urban encampment. To be eligible, individuals had to be experiencing homelessness; be at least 16 years old; and provide informed consent for both the study and the linkage of study data to ICES. Additional information regarding recruitment procedure are available in the *Ku-gaa-gii pimitizi-win* study protocol (33). As our cohort was designed for a different research purpose, we ensured our available sample size was adequate for the main analysis through a dichotomous end-point, independent sample power calculation. Assuming mortality would be 3% in the exposed group and similar to Statistics Canada estimates in the general population control (0.82% in 2021) (36), a sample of 618 individuals was sufficient to assess disparities at 80% power and 0.05 significance, lower than the 640 *Ku-gaa-gii pimitizi-win* participants available to us.

We used the RPDB to generate two additional, unexposed groups as potential matched individuals, in order to assess the presence and extent of disparities in mortality between homelessness and non-homeless populations. The first group, general population housed individuals, were eligible if they were alive and at least 16 years of age as of the earliest potential index date (June 16 2021). Individuals were excluded if they were not residents of the Toronto Census Metropolitan Area (including the census divisions of Durham, York, Toronto and Peel), ineligible for Ontario health insurance in any of 2019, 2020 or 2021, or were already in the exposed group. The second group, low-income housed individuals, had the same eligibility criteria as general population housed group except they had to also reside within a neighborhood in the lowest-income quintile, as determined by Statistics Canada census data, and not already be in the first unexposed group after matching. We included this second matched group to assess the relative importance of homelessness as compared to poverty. In order to control for well-known differences in age, sex and morbidity distribution in these populations that are not necessarily related to homelessness but may significantly impact mortality, we matched these two groups to the exposed group 10 to 1 without replacement by age (+/−5 years), sex-assigned-at-birth (exact) and Charlson comorbidity index category (exact).

### 2.4 Outcomes

The outcome of interest was all-cause mortality within 1 year of the observation start date, defined as any death recorded in the ICES Registered Persons Database.

### 2.5 Covariates

We obtained characteristics for participants in all groups at the start of the 1-year follow-up period. These included age, sex-assigned-at-birth, Charlson comorbidity index category (calculated using hospitalization data from the past year), past diagnosis of hypertension, diabetes, asthma, chronic lung disease, chronic heart disease, history of stroke (within the past 5 years), chronic kidney disease, chronic neurological disorder, liver disease, cancer (within the past 10 years), or HIV/AIDS. Additionally, we measured history of healthcare utilization for mental health or substance use disorders overall and by subgroup (psychotic disorders including schizophrenia, substance use disorders, mood and anxiety disorders, obsessive compulsive disorder/other personality disorders or intentional self-injury). Finally, we measured healthcare utilization in the past year as a proxy for relative acuity, including acute care admissions, emergency department (ED) visits and outpatient visits, as well as history of COVID-19 infection prior to the start of observation. Supplementary material 3 provides detailed information on all covariates used in the study.

### 2.6 Statistical analysis

We present baseline characteristics of study participants by group membership, as well as by outcome status at the end of the follow-up period. *χ*^2^ or analysis of variance (ANOVA) tests were used to compare characteristics between groups, as appropriate. We further provide the number of deaths and mortality rates per 100 person-years of observation in each group overall and stratified by sex-assigned-at-birth and age group. An unadjusted Cox proportional hazards model (using housed individuals as the reference group) estimated the unadjusted mortality hazard ratio with 95% confidence interval. We further present the unadjusted hazard of death through a Kaplan–Meier figure in the Supplementary material. Finally, we estimate the adjusted hazard ratio with 95% confidence interval using multivariable Cox proportional hazards modeling, with adjustment for important confounders that remained unbalanced after matching.

All tests were two-sided with *p* < 0.05 defining statistical significance, and cells <=5 were suppressed to protect patient privacy. All analyses were conducted at ICES using SAS enterprise guide v8.3.

### 2.7 Ethical review

This study received ethics approval from the Research Ethics Board at Unity Health Toronto (REB# 20–272).

## 3 Results

**Figure 1:**
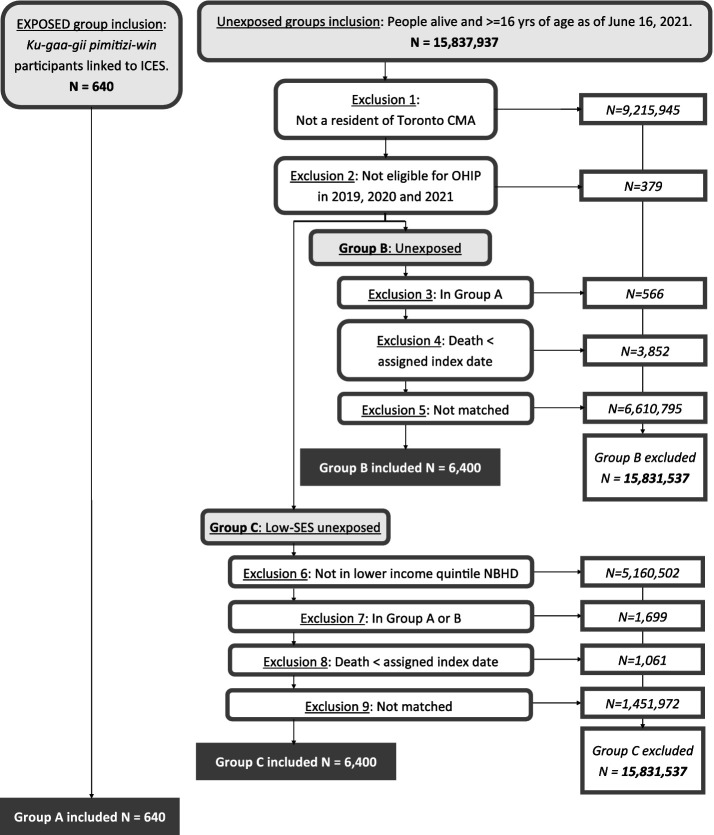
Exposed and unexposed group creation flow diagram, showing numbers included and excluded at each step.

We included 640 participants experiencing homelessness and matched 6,400 individuals for the housed group and 6,400 individuals for the low-income housed group (Figure 1). Characteristics of participants experiencing homelessness successfully linked to ICES were very similar to that of the *Ku-gaa-gii pimitizi-win* study cohort overall (Supplementary Table 1). Following matching, participants experiencing homelessness continued to have significantly higher rates of asthma (20.6% vs. 13.1 and 11.4%), chronic lung disease (14.7% vs. 4.9 and 5.4%), history of stroke (2.7% vs. 1.4 and 1.5%), chronic neurological disorder (4.7% vs. 1.1 and 1.4%), liver disease (6.7% vs. 2.5 and 2.5%), and HIV/AIDS (2.0% vs. 0.4 and 0.7%) (Table 1). They also had substantially higher rates of mental health or substance use disorders as well as healthcare utilization in the past year (all types). Finally, participants experiencing homelessness were significantly more likely to have a history of COVID-19 infection at baseline (17.5% vs. 5.6 and 6.2%).

**Table 1:** Characteristics of the group experiencing homelessness (Group ‘A’) compared to housed individuals (Group ‘B’) and low-income housed individuals (Group ‘C’) at start of observation.

	People experiencing homelessness (Group ‘A’ *n* = 640)	Housed (Group ‘B’ *n* = 6,400)	Low-income housed (Group ‘C’ *n* = 6,400)	*p*-value A vs. B	*p*-value A vs. C
Comorbidities, *N* (%)					
*Hypertension*	117 (18.3%)	1,328 (20.8%)	1,309 (20.5%)	0.14	0.19
*Diabetes*	87 (13.6%)	822 (12.8%)	845 (13.2%)	0.59	0.78
*Asthma*	132 (20.6%)	836 (13.1%)	730 (11.4%)	<0.001	<0.001
*Chronic lung disease*	94 (14.7%)	316 (4.9%)	347 (5.4%)	<0.001	<0.001
*Chronic heart disease*	25 (3.9%)	251 (3.9%)	224 (3.5%)	0.99	0.60
*History of stroke*	17 (2.7%)	89 (1.4%)	96 (1.5%)	0.01	0.03
*Chronic kidney disease*	10 (1.6%)	51 (0.8%)	61 (1.0%)	0.05	0.14
*CND*	30 (4.7%)	70 (1.1%)	90 (1.4%)	<0.001	<0.001
*Liver disease*	43 (6.7%)	157 (2.5%)	160 (2.5%)	<0.001	<0.001
*Cancer*	12 (1.9%)	213 (3.3%)	192 (3.0%)	0.05	0.11
*HIV/AIDS*	13 (2.0%)	23 (0.4%)	44 (0.7%)	<0.001	<0.001
Mental health disorders, *N* (%)					
Any	283 (44.2%)	422 (6.6%)	437 (6.8%)	<0.001	<0.001
*Substance use disorders*	172 (26.9%)	58 (0.9%)	94 (1.5%)	<0.001	<0.001
*Psychotic disorders*	67 (10.5%)	20 (0.3%)	36 (0.6%)	<0.001	<0.001
*Mood/anxiety disorders*	114 (17.8%)	290 (4.5%)	290 (4.5%)	<0.001	<0.001
*OCD/Personality disorders*	21 (3.3%)	8 (0.1%)	6 (0.1%)	<0.001	<0.001
*Intentional self-injury*	31 (4.8%)	8 (0.1%)	9 (0.1%)	<0.001	<0.001
Healthcare in past year, *N* (%)					
*Acute care admissions*					
0	546 (85.3%)	5,899 (92.2%)	5,852 (91.4%)	<0.001	<0.001
1	56 (8.8%)	390 (6.1%)	428 (6.7%)		
2 or more	38 (5.9%)	111 (1.7%)	120 (1.9%)		
*ED visits*					
0	255 (39.8%)	5,282 (82.5%)	5,257 (82.1%)	<0.001	<0.001
1 to 3	211 (33.0%)	1,003 (15.7%)	1,006 (15.7%)		
4 to 6	77 (12.0%)	76 (1.2%)	91 (1.4%)		
7 or more	97 (15.2%)	39 (0.6%)	46 (0.7%)		
History of COVID-19, N (%)	112 (17.5%)	361 (5.6%)	394 (6.2%)	<0.001	<0.001

CND, Chronic Neurological Disorder; OCD, Obsessive compulsive disorder; ED, Emergency Department.

Table 2 presents the number of deaths, rate per 100 person-years and unadjusted mortality hazard ratios (uHRs). A total of 109 participants died over the 1-year follow-up period, 17 PEH, 46 housed, 46 low-income housed. Individuals who died were generally older (mean 60.8 yrs vs. 46.5 among survivors), had higher Charlson comorbidity scores (45.9% had a score of 2+), had any physical comorbidity or a mental health or substance use disorder (though this was driven primarily by participants with substance use disorder [13.8%] or mood/anxiety disorders [15.6%]) or a history of greater healthcare utilization (any type) in the past year (Supplementary Table 2).

**Table 2:** Number, rate per 100 person years and unadjusted hazard ratio of mortality by group membership, overall and stratified by sex-assigned-at-birth or by age group.

	Participants experiencing homelessness			Low-income housed			Housed (Reference)	
	*N* ^1^	Rate/100 person yrs	uHR (95% CI) vs. Group C	*N* ^1^	Rate/100 person yrs	uHR (95% CI) vs. Group C	*N* ^1^	Rate/100 person yrs
Overall	17	2.70	3.72* (2.1–6.5)	46	0.72	1.00 (0.7–1.5)	46	0.72
By sex-assigned at-birth								
*Male*	<=15	2.95	3.91* (2.1–7.6)	30	0.67	0.91 (0.6–1.5)	33	0.74
*Female*	<=5	2.11	NR	16	0.84	NR	13	0.68
By age group^2^								
*16–24 yrs*	<=5	2.32	NR	0	0.00	NR	0	0.00
*25–44 yrs*	<=5	2.04	16.78* (4.0–70.2)	<=5	0.16	1.33 (0.3–6.0)	<=5	0.12
*45–64 yrs*	10	3.72	6.76* (3.0–15.1)	<=25	0.77	1.4 (0.7–2.7)	<=15	0.55
*65+ yrs*	<=5	1.36	0.35 (0.1–2.6)	21	2.88	0.74 (0.4–1.3)	28	3.88
Mean (SD) age at death	47.8 yrs. (13.9)			61.7 yrs. (13.7)			64.8 yrs. (13.4)	

uHR, hazard ratio from an unadjusted Cox proportional hazards model, models used Housed individuals as the reference category; CI, Confidence interval; NR, Cell not reported to avoid recalculating small cells. ^1^Number of deaths. ^2^Age was calculated as [start of observation date] – [participant date of birth, derived from ICES]/365.25, then categorized. *p-value < 0.01.

Overall, participants experiencing homelessness had a mortality rate of 2.7 per 100 person-years, compared to 0.72 in each of the unexposed groups (Table 2). The resulting unadjusted hazard ratio was 3.72 (95% CI 2.1–6.5). Participants experiencing homelessness who died were on average 17 years younger than unexposed individuals. Rates varied between sexes or age groups between 2.0 and 4.0/100 person-years. No deaths were observed among either of the unexposed groups ages 16–24 years (therefore, no hazard ratio could be computed); otherwise, hazard ratios were inversely related to age (reflecting deaths increasing with age among unexposed individuals, but not among PEH), with significant ratios observed for individuals ages 25–44 (16.78 [95% CI 4.0–70.2]) and individuals ages 45–64 years (6.76 [95% CI 3.0–15.1]). No uHRs were significant for the low-income group as compared to housed individuals.

Finally, Table 3 reports the results of the multivariable Cox regression model, estimating hazard ratios for PEH and low income individuals (compared with housed) after adjusting for confounders. We adjusted for age (as a continuous variable, reporting for every additional 10 years of age), number of comorbidities (with zero as the reference), and presence of any mental health or substance use disorder in the model. Participants experiencing homelessness continued to have more than double the hazard of death (2.2 [95% CI 1.21–3.99]) after adjusting for confounding, while low-income housed individuals had no significant difference compared to housed individuals. As expected, age, having at least two comorbidities and presence of any mental health or substance use disorder are also significantly associated with death within 1 year.

**Table 3:** Multivariable Cox proportional hazards model assessing adjusted hazard ratio of group membership and other factors on all-cause mortality over 1-year of follow-up.

	Adjusted HR (95% CI)	*p*-value
Group membership (ref = *Housed individuals*)		
Participants experiencing homelessness	2.20 (1.21–3.99)	0.01
Low-income^1^ housed individuals	0.99 (0.66–1.49)	0.96
Age (every additional 10 yrs)	1.68 (1.43–1.98)	<0.001
Number of comorbidities^2^ (ref = *Zero*)		
1	1.49 (0.77–2.88)	0.23
2 or more	5.42 (3.13–9.40)	<0.001
Any mental health/substance use disorder (ref = *No*)	2.51 (1.58–3.99)	<0.001

CI, Confidence Interval. ^1^Refers to individuals residing in neighborhoods with the lowest income quintile for that region, as determined through Statistics Canada census data. ^2^Comorbidities include the following: hypertension, diabetes, asthma, chronic lung disease, chronic heart disease, history of stroke (within the past 5 years), chronic kidney disease, chronic neurological disorder, liver disease, cancer (within the past 10 years), or HIV/AIDS.

## 4 Discussion

In our representative sample of people experiencing homelessness in Toronto during the COVID-19 pandemic, we found high mortality rates as well as significant excess mortality compared to housed individuals matched on age, sex-assigned-at-birth, Charlson comorbidity score (and low-income, for the low-income housed individuals). Even after further adjusting for number of comorbidities and presence of mental health or substance use disorders, individuals experiencing homelessness had more than twice the mortality hazard ratio of the unexposed group. Notably, people experiencing homelessness were 17 years younger on average at death, which explains why unadjusted models showed that younger age strata have particularly high mortality hazard ratios (For example, a hazard of over 16 for people 25–44 years old).

PEH have long been known to have higher mortality rates than housed individuals (1–5, 7, 37–41). Drivers of excess mortality in this population are many and varied, beginning with structural factors such as extreme poverty (42), racism (42, 43) and colonialism (44, 45) impacting this population at higher rates than housed people. Additionally, PEH as a group have higher than average rates of chronic physical health conditions (43), mental health concerns and substance use (1, 46, 47) [although these two last drivers could also be considered the result of extended homelessness (48)]. Finally, drivers include a host of factors specific to the experience of homelessness itself, such as heightened exposure to environmental hazards, such as extreme temperature and substandard living conditions (47–50); violence, both domestic and interpersonal (42, 45, 51, 52); reduced access to services important to health and well-being, resulting from reduced capacity, lack of communication or transportation options, or lacking identification or documentation (42, 53–55); and stigma resulting in service avoidance or substandard care (55, 56). These factors interact and compound in different ways for each individual experiencing homelessness, and create a significant disparity in mortality between PEH and housed populations as a whole. Thus, our results showing presence of excess mortality are quite consistent with older reports from Toronto and Canada (37, 38), where in the 1990s and early 2000s, mortality rate ratios (MRR) between people experiencing homelessness and housed individuals of varying age groups ranged between 2.3 and 3.7 for men and between 2.1 and 3.1 among women.

However, these age-and sex-stratified older assessments are far less pronounced than the age-specific excess mortality we measured 20 years later, both compared to housed and low-income housed people (among whom we expected disparities to be attenuated on account of the presence of poverty among the unexposed individuals). These findings suggest that although it is almost certainly an important factor, poverty alone is insufficient to explain disparities in short-term mortality experienced by PEH. Furthermore, our findings suggest conditions for people experiencing homelessness in Toronto, particularly for young people ages 16–44 years, have substantially worsened in the past few decades.

Without a local measure of excess mortality immediately preceding the COVID-19 pandemic, we cannot state whether excess mortality has been increasing over the 2000s and 2010s or if the pandemic has caused the observed increase; either or both scenarios are plausible. Past studies in a variety of other settings have shown a steady rise in PEH mortality rate over the 2010s (6, 24, 39, 40) without a concurrent increase in mortality in the general population; however, this observation has not been found consistently in all jurisdictions (41), indicating changes in excess mortality over time may vary from setting to setting. However, one report in San Francisco indicates mortality among PEH has increased during the pandemic (23) and two reports, one from Los Angeles (24) and one using a national US sample (30), shows that the pandemic MRR has increased for PEH compared to pre-pandemic estimates, suggesting the COVID-19 pandemic may also have been associated with some of the observed increase in excess mortality. COVID-19 infection has been identified as one cause contributing to the increase (24, 29, 57), but other concurrent changes, in particular increasing toxicity of drug supply, also play an important role (18, 23, 40, 58).

Finally, our observation that homelessness is a risk factor for mortality independently and in addition to other important overlapping constructs like low-income or presence of mental health or substance use disorders also aligns with past study findings (2, 3, 8, 38). Unlike these other reports, we did not find excess mortality among low-income individuals compared to housed individuals: however, this might be due to the additional matching by Charlson comorbidity score, as well as the shorter observation period in our report.

Our findings extend our understanding of excess mortality among PEH in Canada to the pandemic era, and underscore the urgent need for prevention and intervention strategies aimed at reducing excess mortality among people experiencing homelessness. Because homelessness is a risk factor in addition to (and independently of) low-income and mental health and substance use concerns, stable, affordable housing for all is the ultimate long-term objective policy-makers should target to eliminate excess mortality in this group. In the meantime, a better understanding of the immediate and proximal causes of death in this population is required to target specific issues. In our setting, reliable cause of death information was not available; however, in other settings that examined coroner data, acute drug or alcohol toxicity (23, 24, 46) and traumatic injuries (including transportation related injury) (23, 24) were consistently listed as among the most common causes of death among PEH during the pandemic. This contrasts sharply against top causes of death among housed people, such as heart disease and cancer (59, 60). In both groups, COVID-19 has also become another leading cause of death (23, 24, 57, 59, 60). Each of these causes will require separate strategies for prevention or intervention.

### 4.1 Strengths and limitations

Our study benefits from following a representative cohort of people experiencing homelessness from across Toronto during the pandemic. This provided us with reliable numerators and denominators from which to determine mortality rates, as compared to reports forced to rely on point-in-time counts to estimate the size of the homeless population (a methodologic approach that is known to be problematic) (61), or health or housing administrative data which capture unrepresentative subsections of the population. Meanwhile, our pool of unexposed individuals were sourced from the entire eligible Toronto population, ensuring matches were sourced from a representative group of Torontonians. Furthermore, mortality and other covariate data were derived from reliable administrative data sources, which remove much of the difficulties associated with self-report and follow-up in this population. Finally, our follow-up period was restricted to a one-year window, limiting issues with changes in housing situation confounding results.

However, the following limitations should be noted. First, cause of death information was unavailable for the timeframe of interest. As a result, we could not examine cause of death profiles between groups to identify specific issues that translated to excess death among participants experiencing homelessness. Second, only *Ku-gaa-gii pimitizi win* participants who gave consent and were successfully linked to ICES could be included in this study. While the profile of linked participants is very close to that of participants generally (see Supplementary Table 1), we note that refugees and individuals with ambiguous or temporary status in Canada were somewhat less likely to be included, probably because individuals in these groups are more likely to have no coverage or federal, as opposed to provincial, healthcare coverage, with the result of having no unique identifiers at ICES. Our estimates may thus be somewhat less representative within these subgroups. Finally, and as with most primary research studies, sample size was limited for the purpose of estimating mortality rates within subgroups, leading to relatively large confidence intervals within certain subgroups. While the significance of differences can be reported with confidence, specific rates within subgroups should be interpreted cautiously with these confidence intervals in mind.

## 5 Conclusion

In this representative sample of people experiencing homelessness in Toronto in 2021 and 2022, 1-year all-cause mortality was high and significantly higher than among housed and low-income housed counterparts with similar age, sex-assigned-at-birth and comorbidity level. Homelessness is an important risk factor for mortality, independently of and in addition to poverty or presence of mental health or substance use disorders, and should feature prominently in interventions aiming to improve the lifespan and quality of life of Canadians in the COVID-19 era. Future studies should focus on ascertaining specific causes of mortality among PEH in Toronto to permit development of targeted mortality prevention strategies.

## Data availability statement

Legal data sharing agreements between ICES and its data providers (e.g. healthcare organizations and government) prohibit ICES from making the dataset underlying this analysis publicly available. However, access may be granted to those who meet pre-specified criteria for confidential access, available at www.ices.on.ca/DAS (email: das@ices.on.ca). Requests to review the analytic protocol and code underlying the results presented in this study can also be directed to the Corresponding Author. Requests to access the datasets should be directed to Lucie.Richard@unityhealth.to; DAS@ices.on.ca.

## Ethics statement

The studies involving humans were approved by the Research Ethics Board at Unity Health Toronto. The studies were conducted in accordance with the local legislation and institutional requirements. Written informed consent for participation in this study was provided by the participants’ legal guardians/next of kin.

## Author contributions

LR: Conceptualization, Investigation, Methodology, Project administration, Visualization, Writing – original draft, Writing – review & editing. BC: Formal analysis, Methodology, Software, Writing – review & editing. LW: Writing – original draft, Writing – review & editing. SH: Funding acquisition, Supervision, Validation, Writing – review & editing.
