# Supplementary material for: Disparities in all-cause mortality among people experiencing homelessness in Toronto, Canada during the COVID-19 pandemic: a cohort study
Source: Front Public Health. 2024 Aug 9;12:1401662. doi: 10.3389/fpubh.2024.1401662 (PMC11341496; doi:10.3389/fpubh.2024.1401662)
Supplement: Supplementary file 1 [file Data_Sheet_1.docx]

**Supplement A: The RECORD statement^1^ – checklist of items, extended from the STROBE statement, that should be reported in observational studies using routinely collected health data.**

|  | **Item No.** | **STROBE items** | **Location in manuscript where items are reported** | **RECORD items** | **Location in manuscript where items are reported** |
| --- | --- | --- | --- | --- | --- |
| **Title and abstract** | | | | | |
|  | 1 | (a) Indicate the study’s design with a commonly used term in the title or the abstract (b) Provide in the abstract an informative and balanced summary of what was done and what was found |  | RECORD 1.1: The type of data used should be specified in the title or abstract. When possible, the name of the databases used should be included.  RECORD 1.2: If applicable, the geographic region and timeframe within which the study took place should be reported in the title or abstract.  RECORD 1.3: If linkage between databases was conducted for the study, this should be clearly stated in the title or abstract. | Title; Abstract |
| **Introduction** | | | | | |
| Background rationale | 2 | Explain the scientific background and rationale for the investigation being reported |  |  | Introduction |
| Objectives | 3 | State specific objectives, including any prespecified hypotheses |  |  | Introduction, final paragraph |
| **Methods** | | | | | |
| Study Design | 4 | Present key elements of study design early in the paper |  |  | Methods; Study design and setting |
| Setting | 5 | Describe the setting, locations, and relevant dates, including periods of recruitment, exposure, follow-up, and data collection |  |  | Methods; Study design and setting |
| Participants | 6 | *(a) Cohort study* - Give the eligibility criteria, and the sources and methods of selection of participants. Describe methods of follow-up  *Case-control study* - Give the eligibility criteria, and the sources and methods of case ascertainment and control selection. Give the rationale for the choice of cases and controls  *Cross-sectional study* - Give the eligibility criteria, and the sources and methods of selection of participants  *(b) Cohort study* - For matched studies, give matching criteria and number of exposed and unexposed  *Case-control study* - For matched studies, give matching criteria and the number of controls per case |  | RECORD 6.1: The methods of study population selection (such as codes or algorithms used to identify subjects) should be listed in detail. If this is not possible, an explanation should be provided.  RECORD 6.2: Any validation studies of the codes or algorithms used to select the population should be referenced. If validation was conducted for this study and not published elsewhere, detailed methods and results should be provided.  RECORD 6.3: If the study involved linkage of databases, consider use of a flow diagram or other graphical display to demonstrate the data linkage process, including the number of individuals with linked data at each stage. | 6.1 Methods; Population & Data Sources  6.2 Methods; Population, Covariates; Supplement table 3  6.3 N/A |
| Variables | 7 | Clearly define all outcomes, exposures, predictors, potential confounders, and effect modifiers. Give diagnostic criteria, if applicable. |  | RECORD 7.1: A complete list of codes and algorithms used to classify exposures, outcomes, confounders, and effect modifiers should be provided. If these cannot be reported, an explanation should be provided. | Supplement tables |
| Data sources/ measurement | 8 | For each variable of interest, give sources of data and details of methods of assessment (measurement).  Describe comparability of assessment methods if there is more than one group |  |  | Methods, Data Sources, Population, Outcomes, Covariates, Statistical Analysis |
| Bias | 9 | Describe any efforts to address potential sources of bias |  |  | N/A |
| Study size | 10 | Explain how the study size was arrived at |  |  | N/A (Referred reader to protocol which features sample size calculation) |
| Quantitative variables | 11 | Explain how quantitative variables were handled in the analyses. If applicable, describe which groupings were chosen, and why |  |  | Methods, covariates and Supplement table 3 |
| Statistical methods | 12 | (a) Describe all statistical methods, including those used to control for confounding  (b) Describe any methods used to examine subgroups and interactions  (c) Explain how missing data were addressed  (d) *Cohort study* - If applicable, explain how loss to follow-up was addressed  *Case-control study* - If applicable, explain how matching of cases and controls was addressed  *Cross-sectional study* - If applicable, describe analytical methods taking account of sampling strategy  (e) Describe any sensitivity analyses |  |  | Methods, Statistical Analysis |
| Data access and cleaning methods |  | .. |  | RECORD 12.1: Authors should describe the extent to which the investigators had access to the database population used to create the study population.  RECORD 12.2: Authors should provide information on the data cleaning methods used in the study. | 12.1 Noted work at ICES in Methods, Study Design & Setting  12.2 N/A |
| Linkage |  | .. |  | RECORD 12.3: State whether the study included person-level, institutional-level, or other data linkage across two or more databases. The methods of linkage and methods of linkage quality evaluation should be provided. | Methods, Study Design and Setting |
| **Results** | | | | | |
| Participants | 13 | (a) Report the numbers of individuals at each stage of the study (*e.g.*, numbers potentially eligible, examined for eligibility, confirmed eligible, included in the study, completing follow-up, and analysed)  (b) Give reasons for non-participation at each stage.  (c) Consider use of a flow diagram |  | RECORD 13.1: Describe in detail the selection of the persons included in the study (*i.e.,* study population selection) including filtering based on data quality, data availability and linkage. The selection of included persons can be described in the text and/or by means of the study flow diagram. | 13.1 Figure 1; Results paragraph 1 |
| Descriptive data | 14 | (a) Give characteristics of study participants (*e.g.*, demographic, clinical, social) and information on exposures and potential confounders  (b) Indicate the number of participants with missing data for each variable of interest  (c) *Cohort study* - summarise follow-up time (*e.g.*, average and total amount) |  |  | Results, Paragraph 2 |
| Outcome data | 15 | *Cohort study* - Report numbers of outcome events or summary measures over time  *Case-control study* - Report numbers in each exposure category, or summary measures of exposure  *Cross-sectional study* - Report numbers of outcome events or summary measures |  |  |  |
| Main results | 16 | (a) Give unadjusted estimates and, if applicable, confounder-adjusted estimates and their precision (e.g., 95% confidence interval). Make clear which confounders were adjusted for and why they were included  (b) Report category boundaries when continuous variables were categorized  (c) If relevant, consider translating estimates of relative risk into absolute risk for a meaningful time period |  |  |  |
| Other analyses | 17 | Report other analyses done—e.g., analyses of subgroups and interactions, and sensitivity analyses |  |  |  |
| **Discussion** | | | | | |
| Key results | 18 | Summarise key results with reference to study objectives |  |  | Discussion paragraph 1 |
| Limitations | 19 | Discuss limitations of the study, taking into account sources of potential bias or imprecision. Discuss both direction and magnitude of any potential bias |  | RECORD 19.1: Discuss the implications of using data that were not created or collected to answer the specific research question(s). Include discussion of misclassification bias, unmeasured confounding, missing data, and changing eligibility over time, as they pertain to the study being reported. | Discussion, Limitations section |
| Interpretation | 20 | Give a cautious overall interpretation of results considering objectives, limitations, multiplicity of analyses, results from similar studies, and other relevant evidence |  |  | Discussion, paragraph 1 and 2 |
| Generalisability | 21 | Discuss the generalisability (external validity) of the study results |  |  | Discussion, paragraph 2 and Limitations section |
| **Other Information** | | | | | |
| Funding | 22 | Give the source of funding and the role of the funders for the present study and, if applicable, for the original study on which the present article is based |  |  | Acknowledgements, funding statement |
| Accessibility of protocol, raw data, and programming code |  | .. |  | RECORD 22.1: Authors should provide information on how to access any supplemental information such as the study protocol, raw data, or programming code. |  |

*Checklist is protected under Creative Commons Attribution ([CC BY](http://creativecommons.org/licenses/by/4.0/)) license.

**Supplement B: Description of data sources**

| Data source | Description |
| --- | --- |
| **ICES Registered Persons Database (RPDB)** | The RPDB database provides demographic information on any individual who has ever received an Ontario health card number, including date of birth and death (if applicable), sex-assigned-at-birth, and postal code for each year. |
| **CIHI Discharge Abstract Database (DAD)** | DAD captures administrative (institution-hospital number, admission category, length of stay, disposition), clinical (diagnoses, procedures, physician) and demographic information (patient gender, date of birth, postal code, county and residence code), on hospital discharges including deaths, sign-outs and transfers. |
| **National Ambulatory Care Reporting System (NACRS)** | NACRS captures information on patient visits to hospital and community based ambulatory care such as day surgery, outpatient clinics and emergency departments within Ontario. |
| **Ontario Mental Health Reporting System (OMHRS)** | OHMRS collects data on patients in adult designated inpatient mental health beds. |
| **Ontario Health Insurance Plan (OHIP) claims** | The OHIP claims database contains most claims paid for by the Ontario Health Insurance Plan. The data covers all health care providers who can claim under OHIP (this includes physicians, groups, laboratories, and out-of-province providers) for the purposes of maintaining a record of the patient and physician, services provided, date of the service, associated diagnosis, and fees paid. Excludes services provided by Community Health Centres. |
| **Community Health Centre database (CHC)** | CHC captures services provided in Community Health Centres, which provide primary health and health promotion programs for individuals, families and communities particularly those underserved or who have barriers to accessing other forms of primary care. Provides information on CHC patients, services, physicians and other allied healthcare professionals. |
| **Ontario Cancer Registry (OCR)** | OCR is the provincial database of information for all Ontario residents who have been diagnosed with or who have died of cancer. |
| **Ontario Asthma Database (ASTHMA)** | The Ontario Asthma Database is contains all Ontario asthma patients identified through a validated case definition^2^ since 1991 through primary care patient records and hospital administrative data. |
| **Chronic Obstructive Pulmonary Disease Database (COPD)** | The Ontario Chronic Obstructive Pulmonary Disease Database contains all Ontario COPD patients identified through a validated case definition^3^ since 1991 through primary care patient records and hospital administrative data |
| **Ontario Diabetes Database (ODD)** | The Ontario Diabetes Database contains all Ontario diabetes patients identified through a validated case definition^4^ since 1991 through primary care patient records and hospital administrative data. |
| **Congestive Heart Failure Database (CHF)** | The Ontario Congestive Heart Failure Database contains all Ontario individuals with CHF identified through a validated case definition^5^ since 1991 through primary care patient records and hospital administrative data. |
| **Ontario Hypertension Database (HYPER)** | The Ontario Hypertension Database contains all Ontario individuals with hypertension identified through a validated case definition^6^ since 1991 through primary care patient records and hospital administrative data |
| **Ontario HIV database (HIV)** | The Ontario HIV Database contains all Ontario HIV positive patients identified through a validated case definition^7^ since 1992. |
| **Ontario Dementia database (DEMENTIA)** | The Ontario Dementia Database contains all Ontario individuals with dementia identified through a validated case definition^8^ since 1991. |
|  |  |

**Supplement C – Full variable definitions**

The table below includes all variables included in this analysis (main or supplemental). Included covariates are either shown in existing literature to affect risk for SARS-CoV-2 infection or be related to SARS-CoV-2 related adverse health outcomes (which may also, indirectly, affect behaviours affected risk of infection). In all instances, measures were collected based on a Settler Canadian (Western) understanding of health-related factors and associated prevention guidelines appropriate during the COVID-19 pandemic.

| **Variable** | Definition |
| --- | --- |
| **Age** | Participant’s age as calculated from the ICES Registered Persons Database date of birth and cohort entry date ([start of observation minus date of birth] / 365.25). Expressed numerically or as age groups (‘16 to 29 years’; ‘30 to 49 years’; ‘50 to 69 years’; and ‘70+ years’). |
| **Sex-assigned-at-birth** | Participant’s sex-assigned-at-birth as recorded in the ICES Registered Persons Database. Expressed as ‘Male’ or ‘Female’. |
| **Charlson comorbidity index category** | Participant’s Charlson comorbidity index (calculated using methodology described in Deyo and colleagues,^1^ adapted to ICD-10-CA codes^2^), categorized into ‘No hospitalizations’, ‘0’, ‘1’ or ‘2+’, using hospitalization data from the past year. |
| **Hypertension** | Presence in the ICES Hypertension Database at any point prior to cohort entry. |
| **Diabetes** | Presence in the ICES Diabetes Database at any point prior to cohort entry |
| **Asthma** | Presence in the ICES Asthma Database at any point prior to cohort entry |
| **Chronic lung disease** | Presence in the ICES COPD Database or diagnosis by a physician for chronic lung disease (including emphysema or chronic bronchitis) at any point prior to cohort entry. |
| **Chronic heart disease (heart attack, heart failure, or coronary artery disease)** | Presence in the ICES CHF Database or any hospitalization or physician billing related to coronary artery disease or myocardial infarction at any point prior to cohort entry. Codes include ICD-10-CA code I21, I22, Z955, or T822; CCI procedural code 1IJ50 or 1IJ76; OHIP diagnostic code 410 or 412; or OHIP fee code R741, R742, R743, G298, E646, E651, E652, E654, E655, Z434, Z448. |
| **History of stroke** | One hospitalization or 2 physician billings <1 yr apart within the past 5 years for healthcare related to a past stroke. Codes include ICD-10-ca codes I60, I61, I63, I64, H341, G450, G451, G452, G453, G458, G459, or H340; or OHIP diagnostic code 436, 432 or 435. |
| **Chronic kidney disease** | One hospitalization or 2 physician billings or ED visits <1 yr apart within the past year for healthcare related to chronic kidney disease, including ICD-10-CA codes E102, E112, E132, E142, I12, I13, N08, N18, or N19; or OHIP diagnostic code 403. |
| **Chronic neurological disorder** | Presence in the Dementia database; or, any hospitalization, ED visit or physician billing in the past year relating to traumatic brain injury or Guillaine-Barre Syndrome; or, one hospitalization or at least 3 physician billings in the past year related to epilepsy; or, one hospitalization or at least 5physician billings over the past 2 years related to multiple sclerosis. Codes relating to traumatic brain injury include ICD-10-CA code F072, S020, S021, S023, S027, S028, S029, S06, S071, T902, or T905. Codes relating to epilepsy include ICD-10-CA code G40, G410, G411, G412, G418, G419, R560, R568, F445, or F803; or OHIP diagnostic code 345. Codes relating to Guillaine-Barre syndrome include ICD-10-CA code G610. Codes relating to multiple sclerosis include ICD-10-CA code G25; or OHIP diagnostic code 340. |
| **Liver disease** | Any hospitalization, ED visit or physician billing in the past year for healthcare related to liver disease, including ICD-10-CA codes K70, K713, K714, K715, K717, K721, K729, K73, K74, K753, K754, K758, K759, K76, K77, B16, B17, B18, B19, B942, E831, E830, R160, R162, R18, or I85; OHIP diagnostic codes 571, 573 or 070; or OHIP fee codes Z551 or Z554. |
| **Cancer** | Diagnosis in the Ontario Cancer Registry within 10 years of cohort entry. |
| **HIV/AIDS** | Presence in the ICES HIV Database at any point prior to cohort entry. |
| **Any mental health disorder** | One hospitalization or at least 3 outpatient/ED visits in the past year for any mental health or substance use related concern. |
| **Substance use disorder** | One hospitalization or at least 3 outpatient/ED visits in the past year for substance use related concern. |
| **Psychotic disorders including schizophrenia** | One hospitalization or at least 3 outpatient/ED visits in the past year for psychotic disorder related concern, including schizophrenia. |
| **Mood/anxiety disorder** | One hospitalization or at least 3 outpatient/ED visits in the past year for mood or anxiety related concern |
| **OCD/personality disorder** | One hospitalization or at least 3 outpatient/ED visits in the past year for obsessive compulsive disorder or other personality disorder related concern |
| **Intentional self-injury** | Any hospitalization or ED visit in the past year related to self-harm or intentional self-injury |
| **Acute care admissions in the past year** | Number of acute care admissions in the past year before cohort entry. Categorized into None, 1 or 2+ admissions |
| **ED visits in the past year** | Number of emergency department visits in the past year before cohort entry. Categorized into None, 1-3, 4-6 and 7+ visits |
| **History of COVID-19 infection** | History of any COVID-19 infection at cohort entry. |

**Supplement D: Supplementary tables and figures**

**Supplement table 1: Characteristics of the *Ku-gaa-gii pimitizi-win* cohort overall and *Ku-gaa-gii pimitizi-win* participants successfully linked to ICES**

|  | **Total (N=736)** | **Linked at ICES (N=640)** |
| --- | --- | --- |
| Age category, N (%) |  |  |
| 16 to 29 years | 100 (13.59%) | 74 (11.56%) |
| 30 to 49 years | 326 (44.29%) | 280 (43.75%) |
| 50 to 69 years | 263 (35.73%) | 242 (37.81%) |
| 70+ years | 47 (6.39%) | 44 (6.88%) |
| Sex-assigned-at-birth, N (%) |  |  |
| Male | 496 (67.39%) | 449 (70.16%) |
| Female | 236 (32.07%) | 188 (29.38%) |
| Other/Refused/Don’t know | 4 (0.55%) | <=5 |
| Self-identify as Indigenous, N (%) | 76 (10.23%) | 72 (11.25%) |
| Race category, N (%) |  |  |
| White | 359 (48.78%) | 332 (51.88%) |
| Black | 159 (21.60%) | 122 (19.06%) |
| Indigenous | 28 (3.80%) | 28 (4.38%) |
| Other/multiracial | 157 (21.33%) | 134 (20.94%) |
| Refused/Don't know | 33 (4.48%) | 24 (3.75%) |
| Citizenship status, N (%) |  |  |
| Citizen | 564 (76.63%) | 520 (81.25%) |
| Landed immigrant/Permanent Resident | 90 (12.23%) | 84 (13.13%) |
| Refugee claimant | 55 (7.47%) | 24 (3.75%) |
| Temporary/Other | 20 (2.72%) | <=10 |
| Refused/Don't know | 7 (0.95%) | <=5 |
| Immigrated to Canada within the past 10 years, N (%) |  |  |
| N/A (born in Canada) | 441 (59.92%) | 404 (63.13%) |
| No, immigrated > 10 years ago | 187 (25.41%) | 172 (26.88%) |
| Yes, immigrated <=10 years ago | 108 (14.67%) | 64 (10.0%) |
| Highest level of education completed, N (%) |  |  |
| Have not completed High School | 208 (28.26%) | 188 (29.38%) |
| High School or Secondary School | 255 (34.65%) | 223 (34.84%) |
| Any post-secondary | 264 (35.87%) | 223 (34.84%) |
| Refused/Don't know | 9 (1.22%) | 6 (0.94%) |
| BMI category, N (%) |  |  |
| Underweight/Normal | 339 (46.06%) | 301 (47.03%) |
| Overweight | 227 (30.84%) | 199 (31.09%) |
| Obese | 141 (19.16%) | 119 (18.59%) |
| Missing | 29 (3.94%) | 21 (3.28%) |
| Confidence of interviewer in responses received during interview, N (%) |  |  |
| Confident | 715 (97.15%) | 623 (97.34%) |
| Unconfident | 12 (1.63%) | 9 (1.41%) |
| Very unconfident | 9 (1.22%) | 8 (1.25%) |

**Supplement table 2: Characteristics of the cohort overall, by outcome (died before end of follow-up vs no death prior to end of follow-up)**

|  | **Total (N=13,440)** | **No outcome (n=13,331)** | **All-cause mortality (n=109)** | ***p-value*** |
| --- | --- | --- | --- | --- |
| Age, mean (SD) | 46.6 (14.4) | 46.5 (14.4) | 60.8 (14.7) | <.001 |
| Age, median (IQR) | 46 (36-57) | 46 (36-57) | 63 (50-69) | <.001 |
| Male-at-birth, N (%) | 9,408 (70.0%) | 9,332 (70.0%) | 76 (69.7%) | 0.95 |
| Charlson score category, N (%) |  |  |  |  |
| *No hospitalizations* | 11,193 (83.3%) | 11,150 (83.6%) | 43 (39.4%) | <.001 |
| *0* | 1,344 (10.0%) | 1,332 (10.0%) | 12 (11.0%) |  |
| *1* | 399 (3.0%) | 392 (2.9%) | 7 (6.4%) |  |
| *2+* | 504 (3.8%) | 457 (3.4%) | 47 (43.1%) |  |
| Comorbidities, N (%) |  |  |  |  |
| *Hypertension* | 2,754 (20.5%) | 2,698 (20.2%) | 56 (51.4%) | <.001 |
| *Diabetes* | 1,754 (13.1%) | 1,715 (12.9%) | 39 (35.8%) | <.001 |
| *Asthma* | 1,698 (12.6%) | 1,673 (12.5%) | 25 (22.9%) | 0.001 |
| *Chronic Lung Disease* | 757 (5.6%) | 724 (5.4%) | 33 (30.3%) | <.001 |
| *Chronic Heart Disease* | 500 (3.7%) | 475 (3.6%) | 25 (22.9%) | <.001 |
| *History of Stroke* | 202 (1.5%) | 191 (1.4%) | 11 (10.1%) | <.001 |
| *Chronic Kidney Disease* | 122 (0.9%) | 110 (0.8%) | 12 (11.0%) | <.001 |
| *CND* | 190 (1.4%) | 175 (1.3%) | 15 (13.8%) | <.001 |
| *Liver Disease* | 360 (2.7%) | 346 (2.6%) | 14 (12.8%) | <.001 |
| *Cancer* | 417 (3.1%) | 383 (2.9%) | 34 (31.2%) | <.001 |
| *HIV/AIDS* | 80 (0.6%) | <=80 | <=5 | 0.003 |
| Mental health or substance use, N (%) | 1,142 (8.5%) | 1,113 (8.3%) | 29 (26.6%) | <.001 |
| *Substance use disorders* | 324 (2.4%) | 309 (2.3%) | 15 (13.8%) | <.001 |
| *Psychotic disorders* | 123 (0.9%) | <=130 | <=5 | 1 |
| *Mood/anxiety disorders* | 694 (5.2%) | 677 (5.1%) | 17 (15.6%) | <.001 |
| *OCD/Personality disorders* | 35 (0.3%) | 35 (0.3%) | 0 (0.0%) | 0.59 |
| *Intentional self-injury* | 48 (0.4%) | 48 (0.4%) | 0 (0.0%) | 0.53 |
| Hospitalizations in past year, N (%) |  |  |  |  |
| 0 | 12,297 (91.5%) | 12,242 (91.8%) | 55 (50.5%) | <.001 |
| 1 | 874 (6.5%) | 848 (6.4%) | 26 (23.9%) |  |
| 2 or more | 269 (2.0%) | 241 (1.8%) | 28 (25.7%) |  |
| ED visits in past year, N (%) |  |  |  |  |
| 0 | 10,794 (80.3%) | 10,757 (80.7%) | 37 (33.9%) | <.001 |
| 1 to 3 | 2,220 (16.5%) | 2,173 (16.3%) | 47 (43.1%) |  |
| 4 to 6 | 244 (1.8%) | 227 (1.7%) | 17 (15.6%) |  |
| 7 or more | 182 (1.4%) | 174 (1.3%) | 8 (7.3%) |  |
| History of COVID-19, N (%) | 867 (6.5%) | 855 (6.4%) | 12 (11.0%) | 0.05 |

CND=Chronic Neurological Disorder; OCD=Obsessive-compulsive disorder; ED=Emergency Department

**Supplement Figure 1: Cumulative Incidence of all-cause death over 1 year of follow-up, by group membership (exposed homeless individuals [Group ‘A’], unexposed housed controls [Group ‘B’], and unexposed low-income housed controls [Group ‘C’])**


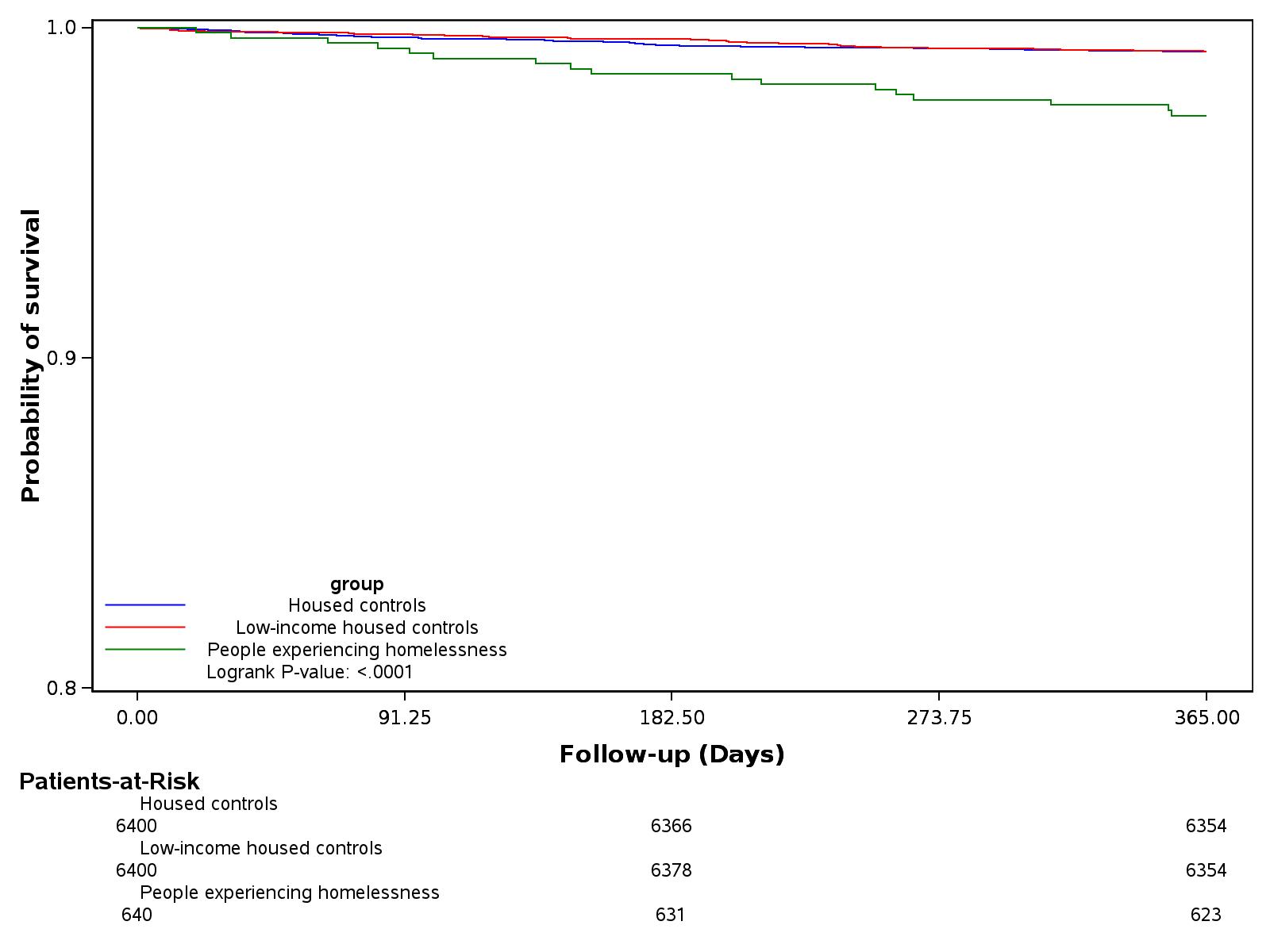


**References**

1. Benchimol EI, Smeeth L, Guttmann A, Harron K, Moher D, Petersen I, Sørensen HT, von Elm E, Langan SM, RECORD Working Committee. The REporting of studies Conducted using Observational Routinely-collected health Data (RECORD) statement. *PLoS medicine*. 2015 Oct 6;12(10):e1001885.
2. Gershon AS, Wang C, Guan J, Vasilevska-Ristovska J, Cicutto L, To T. Identifying patients with physician-diagnosed asthma in health administrative databases. *Canadian Respiratory Journal*. 2009 Nov 1;16:183-8.
3. Gershon AS, Wang C, Guan J, Vasilevska-Ristovska J, Cicutto L, To T. Identifying individuals with physcian diagnosed COPD in health administrative databases. *COPD: Journal of Chronic Obstructive Pulmonary Disease*. 2009 Jan 1;6(5):388-94.
4. Guttmann A, Nakhla M, Henderson M, To T, Daneman D, Cauch‐Dudek K, Wang X, Lam K, Hux J. Validation of a health administrative data algorithm for assessing the epidemiology of diabetes in Canadian children. *Pediatric diabetes*. 2010 Mar;11(2):122-8.
5. Schultz SE, Rothwell DM, Chen Z, Tu K. Identifying cases of congestive heart failure from administrative data: a validation study using primary care patient records. *Chronic diseases and injuries in Canada*. 2013 Jun 1;33(3).
6. Tu K, Campbell NR, Chen ZL, Cauch-Dudek KJ, McAlister FA. Accuracy of administrative databases in identifying patients with hypertension. *Open medicine*. 2007;1(1):e18.
7. Antoniou T, Zagorski B, Loutfy MR, Strike C, Glazier RH. Validation of case-finding algorithms derived from administrative data for identifying adults living with human immunodeficiency virus infection. *PloS one*. 2011 Jun 30;6(6):e21748.
8. Jaakkimainen RL, Bronskill SE, Tierney MC, Herrmann N, Green D, Young J, Ivers N, Butt D, Widdifield J, Tu K. Identification of physician-diagnosed Alzheimer’s disease and related dementias in population-based administrative data: a validation study using family physicians’ electronic medical records. *Journal of Alzheimer's Disease*. 2016 Jan 1;54(1):337-49.
9. Deyo RA, Cherkin DC, Ciol MA. Adapting a clinical comorbidity index for use with ICD-9-CM administrative databases. *J Clin Epidemiol* 1992;45:613-9
10. Sundararajan V, Henderson T, Perry C, et al. New ICD-10 version of the Charlson comorbidity index predicted in-hospital mortality. *J Clin Epidemiol* 2004;57:1288-94.
